# Functional and Radiological Outcomes of Proximal Tibia Fracture Treated With Dual Tibial Plating: A Retrospective Study

**DOI:** 10.7759/cureus.105443

**Published:** 2026-03-18

**Authors:** Amit Kumar Varun, Gils Thampi, Nagakumar J S, Manoj K Ramachandraiah

**Affiliations:** 1 Orthopaedics, Sri Devaraj Urs Medical College (SDUMC), Kolar, IND

**Keywords:** bicondylar fracture, dual plating, proximal tibial fracture, rasmussen score, rust score

## Abstract

Introduction: Proximal tibial fractures, particularly bicondylar variants (AO/OTA 41-C), are complex injuries usually resulting from high-energy trauma. These fractures are characterized by articular comminution and metaphyseal instability. Dual tibial plating provides superior biomechanical stability by stabilizing both columns, which reduces the risk of varus collapse and facilitates early mobilization compared to isolated lateral plating.

Materials and methods: This retrospective observational study included 50 skeletally mature patients (≥18 years) with closed or Gustilo-Anderson type I or II proximal tibial fractures treated with dual tibial plating at a tertiary care center between July 2022 and June 2025. Patients with type III open fractures, pathological fractures, polytrauma affecting rehabilitation, or incomplete records were excluded. Clinical outcomes were assessed using the visual analog scale (VAS) for pain and knee range of motion (ROM). Radiological union was evaluated using the radiographic union score for tibial fractures (RUST) at 1, 3, and 6 months. Statistical analysis was performed using IBM Corp. Released 2020. IBM SPSS Statistics for Windows, Version 26. Armonk, NY: IBM Corp., with p < 0.05 considered statistically significant.

Results: All 50 patients completed a minimum follow-up period of six months. Clinical union was achieved in all cases. Radiological union was confirmed in 48 patients (96%) at the final follow-up, while delayed union occurred in two patients (4%). Superficial infection was observed in four patients (8%), with no cases of deep infection reported. Significant improvements in VAS pain scores and knee range of motion were observed during serial follow-ups (p < 0.001).

Conclusion: Dual tibial plating offers stable and reliable fixation for complex proximal tibial fractures, leading to high union rates, favorable functional recovery, and an acceptable complication profile. Therefore, it is an effective treatment option for appropriately selected patients.

## Introduction

Proximal tibial fractures are complex periarticular injuries that frequently result from road traffic accidents and falls from heights [1]. Bicondylar fractures, involving both the medial and lateral columns, are associated with instability, soft-tissue damage, and challenges in maintaining alignment, often complicating surgical decision-making [2].

The goals of management include restoring the articular surface, maintaining the mechanical axis, and promoting early mobilization to prevent stiffness and functional impairment while preserving long-term joint function [3]. Isolated lateral plating may be insufficient for fractures involving medial column comminution, as it predisposes the patient to varus collapse and fixation failure, particularly in high-energy injury patterns [4].

Dual tibial plating stabilizes both columns, providing superior biomechanical strength and increased resistance to axial and varus forces [5]. Advances in surgical techniques and soft-tissue management have resulted in acceptable complication rates, supporting its use in complex fracture patterns [6]. This study evaluates the functional and radiological outcomes of proximal tibial fractures treated with dual tibial plating.

## Materials and methods

This retrospective study was conducted at RL Jalappa Hospital and Research Centre, Kolar, following approval from the Institutional Ethics Committee (SDUAHER/R&D/CEC/SDUMC-PG/313/NF/-2025-26). Medical records of patients treated between July 2022 and June 2025 were reviewed to collect demographic, clinical, and radiological outcome data according to standardized institutional follow-up protocols.

Inclusion criteria comprised skeletally mature patients with AO/OTA 41-C proximal tibial fractures treated with dual tibial plating during the study period. The study cohort consisted of consecutive eligible patients identified from institutional records. Exclusion criteria included pathological fractures, polytrauma affecting postoperative rehabilitation, Gustilo-Anderson type III open fractures, associated neurovascular injuries, previous surgery involving the affected tibia, metabolic bone disease, active infection around the knee joint, and incomplete clinical or radiological follow-up. A total of 50 consecutive patients who met these eligibility criteria were included in the final analysis.

At presentation, all patients underwent a standard trauma evaluation, including a clinical assessment and radiographic imaging with anteroposterior and lateral knee radiographs. Computed tomography (CT) scans were reviewed when available as part of the retrospective record analysis. Multiplanar CT imaging and three-dimensional reconstructions were utilized in cases where CT imaging had been performed, aiding in fracture classification, assessment of articular comminution, and preoperative surgical planning. The AO/OTA fracture classification was confirmed using the available radiographic and CT imaging data.

Initial management focused on limb stabilization, pain control, and soft-tissue assessment in accordance with institutional trauma protocols. In patients presenting with significant soft-tissue swelling or high-energy injury patterns, temporary knee-spanning external fixation was employed as part of a staged management approach to maintain limb alignment and facilitate soft-tissue recovery. Definitive internal fixation with dual plating was performed once swelling subsided and the soft tissues were deemed suitable for surgery, typically within 5 to 10 days following initial stabilization.

Definitive fixation was performed using a medial 3.5-mm locking plate and a lateral 4.5-mm proximal tibial locking compression plate (DePuy Synthes, Oberdorf, Switzerland). Patients were positioned supine on a radiolucent operating table under fluoroscopic guidance. Fracture reduction was achieved using indirect reduction techniques with temporary fixation as needed. Dual plating was performed through separate medial and anterolateral surgical approaches, prioritizing the restoration of articular alignment and the preservation of surrounding soft tissues. The sequence of fixation was determined based on fracture configuration and intraoperative reduction requirements. All procedures were performed by or under the direct supervision of senior orthopedic trauma surgeons experienced in periarticular fracture fixation. Surgical planning, reduction techniques, and fixation strategies were standardized according to fracture morphology and soft-tissue status.

Perioperative antibiotic prophylaxis was administered according to the institutional protocol, typically involving intravenous antibiotics given before the incision and continued postoperatively for a short duration as indicated.

Postoperatively, patients followed a standardized rehabilitation protocol. Passive and active-assisted knee range-of-motion exercises were initiated within the first postoperative week, as tolerated, to minimize joint stiffness. Gradual knee mobilization was encouraged under physiotherapy supervision. Partial weight-bearing was generally permitted at approximately six weeks, depending on fracture stability and radiographic findings, while full weight-bearing was allowed between 10 and 12 weeks once radiological evidence of fracture union was observed.

Patients were followed up at one, three, and six months postoperatively. Clinical assessments included pain evaluation using the visual analog scale and measurement of knee range of motion. Radiological assessments were conducted using standard anteroposterior and lateral radiographs. Immediate postoperative radiographs were also reviewed to assess the quality of articular reduction. A residual articular step-off or gap was evaluated on postoperative radiographs and categorized as an anatomical (<2 mm), acceptable (2-5 mm), or poor (>5 mm) reduction.

Fracture healing was evaluated using the radiographic union score for tibial fractures (RUST), as described by Whelan et al. [7]. The RUST score assigns a value from 1 to 3 to each of the four cortices based on callus formation and fracture line visibility, resulting in a total score ranging from 4 (no healing) to 12 (complete union). Higher scores indicate more advanced cortical bridging. In this study, a RUST score of ≥9 was considered indicative of radiological union.

Follow-up radiographs were independently reviewed by two orthopedic surgeons experienced in trauma surgery who were not involved in the initial surgical procedures. The reviewers were blinded to the patients' clinical and functional outcomes during the radiographic evaluation. In cases of disagreement in RUST scoring, a consensus review was conducted to determine the final score.

Functional outcomes were assessed using the Rasmussen functional score, originally described by Rasmussen [8]. This scoring system evaluates pain, walking capacity, range of motion, and stability, with a maximum score of 30 points. Scores were categorized as excellent (27-30), good (20-26), fair (10-19), and poor (<10). Functional outcome assessments were conducted based on clinical follow-up records by an orthopedic surgeon who was not involved in the initial surgical procedures.

Statistical analysis

Data were analyzed using IBM Corp. Released 2020. IBM SPSS Statistics for Windows, Version 26. Armonk, NY: IBM Corp. Continuous variables are presented as mean ± standard deviation, while categorical variables are expressed as frequencies and percentages. Preoperative and postoperative visual analog scale scores and knee range of motion were compared using paired t-tests. A p-value of less than 0.05 was considered statistically significant.

## Results

All 50 patients completed a minimum follow-up period of six months. Clinical union was achieved in all cases, and radiological union was confirmed in 48 patients (96%) at the final follow-up.

The cohort had a mean age of 42 ± 11 years, with a male predominance (39 patients, 78%). The distribution of the affected side was nearly equal, with 28 patients (56%) sustaining right-sided injuries and 22 patients (44%) sustaining left-sided injuries. All fractures were classified as AO/OTA 41-C bicondylar patterns, indicating a relatively uniform high-energy injury population without significant demographic variability influencing outcome measures (Table 1).

**Table 1:** Patient demographics and fracture characteristics (n = 50) AO/OTA: Arbeitsgemeinschaft für Osteosynthesefragen/Orthopaedic Trauma Association classification. Values are presented as mean ± standard deviation or number (percentage).

Variable	Result
Age (years), mean ± SD	42 ± 11
Sex – Male, n (%)	39 (78%)
Sex – Female, n (%)	11 (22%)
Side of injury – Right, n (%)	28 (56%)
Side of injury – Left, n (%)	22 (44%)
AO/OTA 41-C bicondylar fractures, n (%)	50 (100%)

Mean VAS pain scores decreased significantly from 8.2 ± 1.1 preoperatively to 4.5 ± 1.2 at one month (t = 19.84, p < 0.001), 2.8 ± 0.9 at three months (t = 29.67, p < 0.001), and 1.5 ± 0.7 at six months (t = 38.92, p < 0.001). This represents an approximate 82% reduction in pain severity by the final follow-up, indicating sustained and clinically meaningful symptomatic improvement following dual tibial plating (Table 2).

**Table 2:** Pain severity was assessed by visual analog scale over follow-up (n=50) * Compared with preoperative values using a paired t-test. VAS: Visual analog scale (0–10). Values are expressed as mean ± standard deviation. t-values represent paired t-test comparisons with preoperative values (degrees of freedom = 49).

Time Point	Mean VAS ± SD	t-value*	p-value
Preoperative	8.2 ± 1.1	–	–
1 month	4.5 ± 1.2	19.84	<0.001
3 months	2.8 ± 0.9	29.67	<0.001
6 months	1.5 ± 0.7	38.92	<0.001

Knee range of motion improved significantly during the follow-up period. Mean knee flexion increased from 45° ± 8° preoperatively to 85° ± 10° at one month (t = 22.31, p < 0.001), 110° ± 9° at three months (t = 34.56, p < 0.001), and 125° ± 7° at six months (t = 42.87, p < 0.001). Concurrently, mean extension lag decreased from 15° ± 4° preoperatively to 8° ± 3° (t = 13.72, p < 0.001), 3° ± 2° (t = 21.48, p < 0.001), and 2° ± 1° (t = 24.96, p < 0.001) at the corresponding time points. These results demonstrate a statistically significant and progressive functional recovery following dual tibial plating (Table 3).

**Table 3:** Knee range of motion recovery (n=50) * Compared with preoperative ROM using a paired t-test. Values are expressed as mean ± standard deviation. The t-values represent paired t-test comparisons with preoperative measurements (degrees of freedom = 49).

Follow-up	Mean Knee Flexion (°) ± SD	t-value*	p-value	Mean Extension Lag (°) ± SD	t-value*	p-value
Preoperative	45 ± 8	–	–	15 ± 4	–	–
1 month	85 ± 10	22.31	<0.001	8 ± 3	13.72	<0.001
3 months	110 ± 9	34.56	<0.001	3 ± 2	21.48	<0.001
6 months	125 ± 7	42.87	<0.001	2 ± 1	24.96	<0.001

RUST scores increased from 8.4 at 1 month to 11.4 at three months, reaching a maximum score of 12 by six months, confirming progressive cortical bridging and consolidation. Radiological union was achieved in 96% of cases (Table 4).

**Table 4:** Radiological healing assessed by RUST score (n=50) RUST: Radiographic Union Score for Tibia (maximum score 12)

Follow-up	Mean RUST Score ± SD	Interpretation
1 month	8.4 ± 1.1	Early callus formation
3 months	11.4 ± 0.9	Advanced cortical bridging
6 months	12.0 ± 0.0	Complete radiological union

Radiological union was achieved in 96% of patients, with delayed union occurring in 4%. Superficial infections were observed in 8% of cases but resolved with treatment. No deep infections were reported. The mean Rasmussen score of 28.2 indicates predominantly excellent functional outcomes (Table 5).

**Table 5:** Complications and functional outcome at final follow-up (n=50) Rasmussen score assesses functional knee outcome (maximum score 30)

Parameter	Number (%)
Radiological union	48 (96%)
Delayed union	2 (4%)
Superficial infection	4 (8%)
Deep infection	0 (0%)
Mean Rasmussen functional score	28.2 ± 1.4 / 30

Immediate postoperative radiographic evaluation demonstrated anatomical reduction (<2 mm residual step-off) in 36 patients (72%), acceptable reduction (2-5 mm) in 11 patients (22%), and poor reduction (>5 mm) in 3 patients (6%) (Table 6).

**Table 6:** Immediate postoperative articular reduction quality (n=50) Articular reduction quality was assessed on immediate postoperative radiographs based on residual articular step-off or gap

Reduction Quality	Number of Patients (n=50)	Percentage (%)
Anatomical (<2 mm step-off)	36	72
Acceptable (2–5 mm step-off)	11	22
Poor (>5 mm step-off)	3	6

Representative case

**Figure 1:**
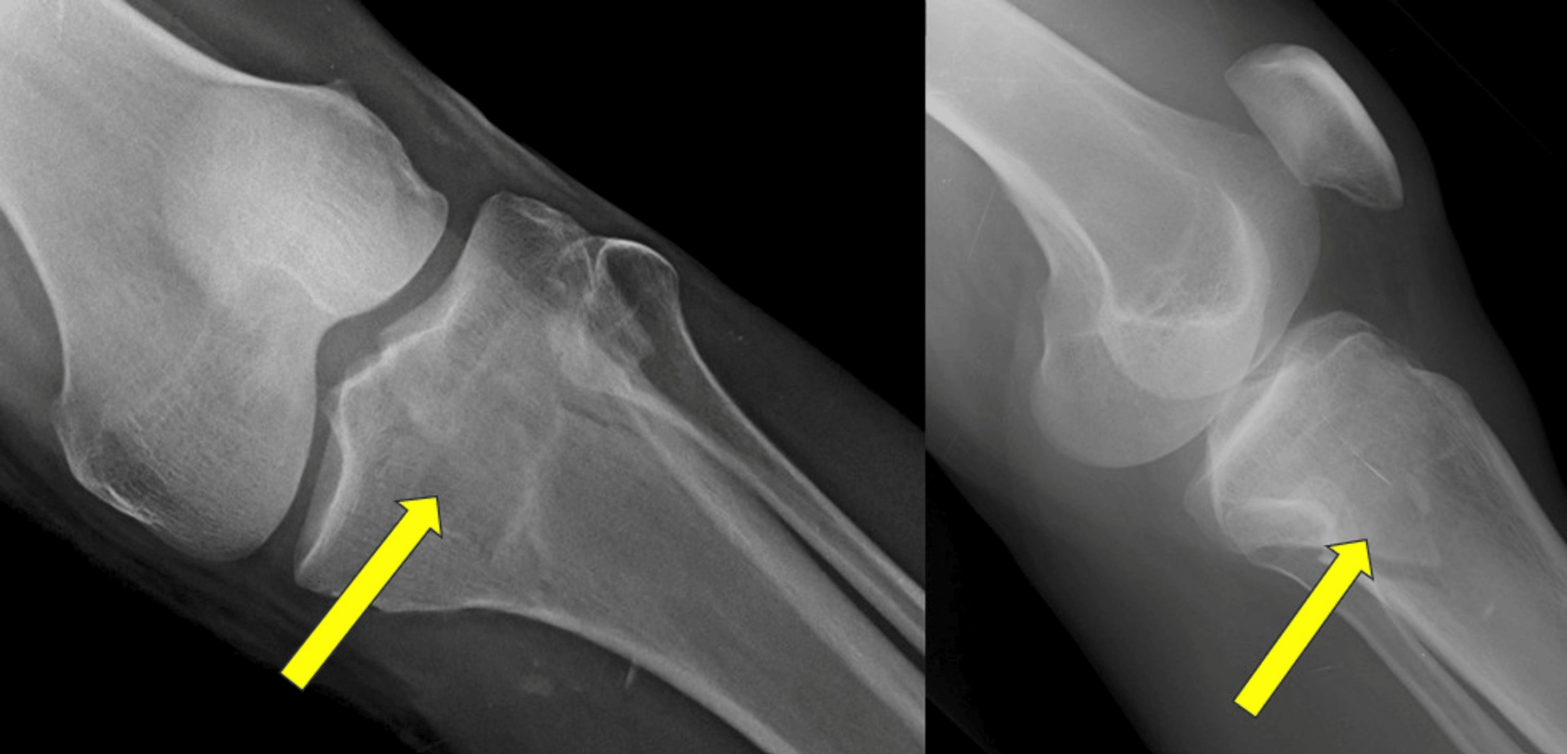
Preoperative anteroposterior and lateral radiographs demonstrating bicondylar proximal tibial fracture

**Figure 2:**
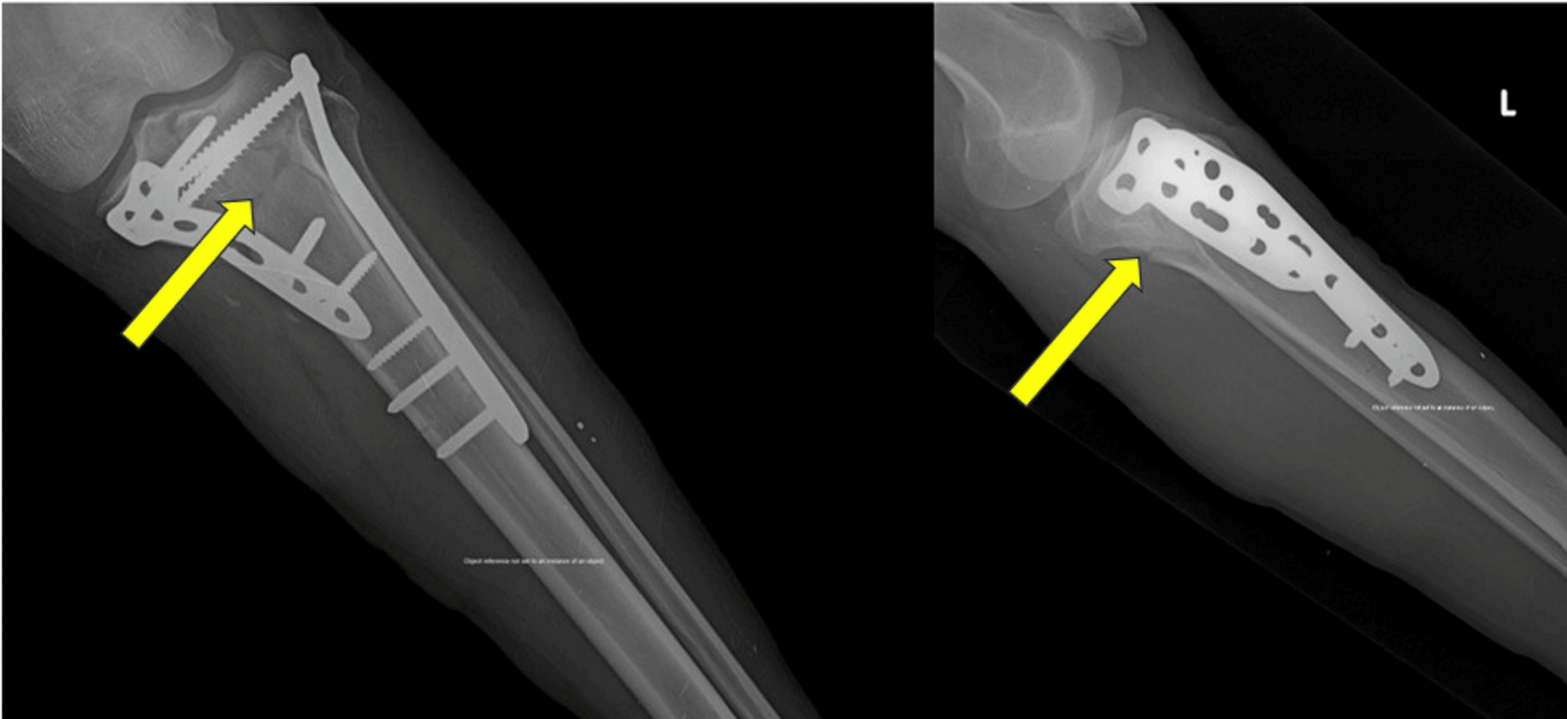
Immediate postoperative radiographs showing dual medial and lateral plate fixation with satisfactory fracture reduction

**Figure 3:**
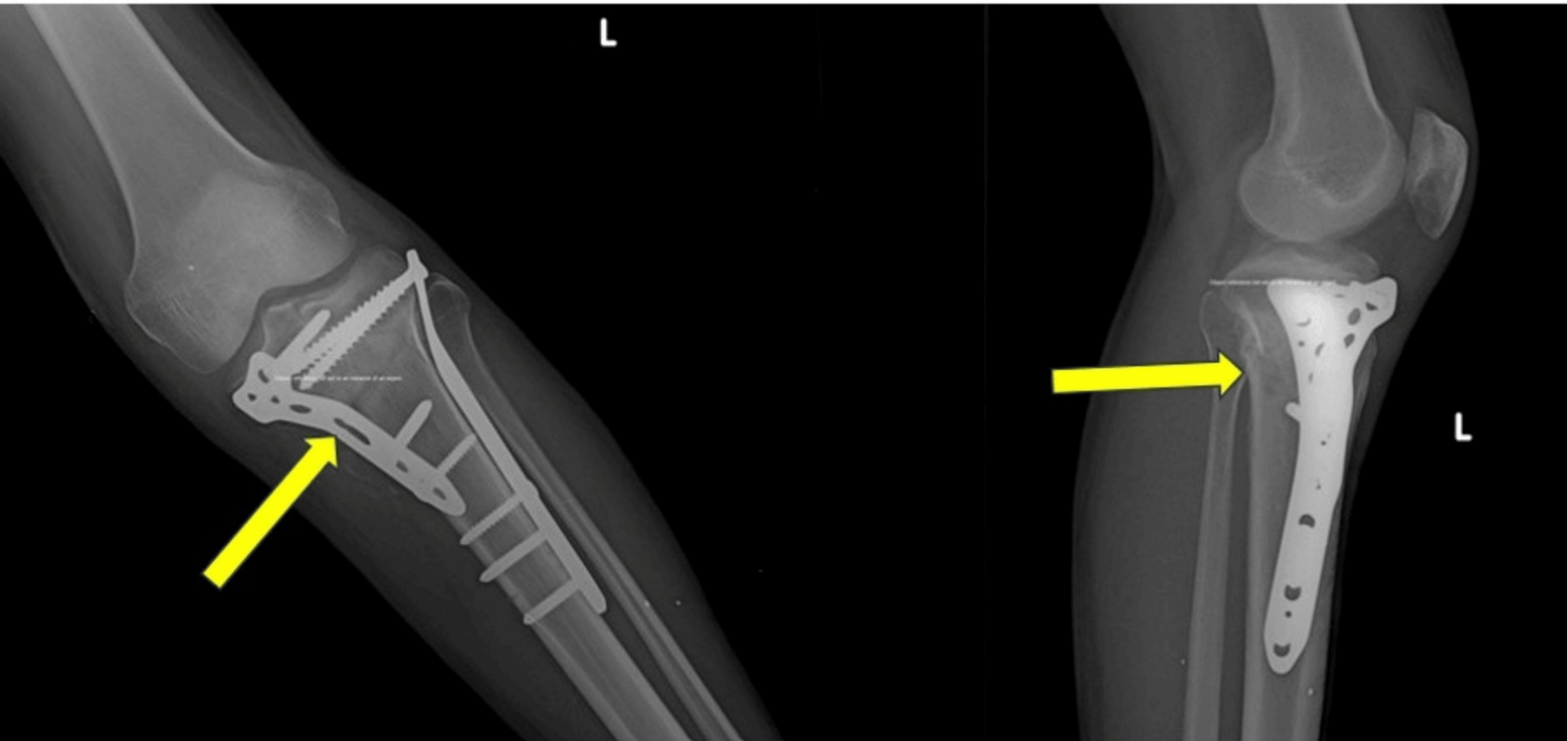
Final follow-up radiographs at six months demonstrating fracture union and maintained alignment

A representative case from the study cohort is illustrated in Figures 1-3. A 42-year-old male presented with a bicondylar left proximal tibial fracture following a road traffic accident. Figure 1 shows the preoperative anteroposterior and lateral radiographs, demonstrating the bicondylar left proximal tibial fracture. The fracture was managed surgically using dual plating with medial and lateral locking plates. Figure 2 displays the immediate postoperative radiographs, showing satisfactory fracture reduction and stable fixation. At the six-month follow-up, Figure 3 demonstrates complete fracture union with restoration of joint alignment and satisfactory functional recovery.

## Discussion

Dual tibial plating has emerged as a reliable fixation strategy for bicondylar proximal tibial fractures due to its ability to restore stability and maintain alignment [9]. In the present study, high union rates and excellent functional outcomes were observed, comparable to those reported in previous series [10-12]. The consistent radiological consolidation and satisfactory Rasmussen scores further support the effectiveness of this technique in managing complex periarticular fracture patterns. These findings reinforce the growing consensus favoring dual-column stabilization in unstable bicondylar injuries and underscore its role as a dependable surgical option for appropriately selected patients.

Biomechanical studies have demonstrated that dual plating offers superior resistance to axial and varus forces compared to single lateral plating, particularly in fractures with medial column deficiencies [13,14]. This biomechanical advantage likely contributed to the low incidence of malalignment and implant failure observed in this study. By stabilizing both columns, dual plating enhances construct rigidity and distributes mechanical stress more evenly across the fracture site, thereby maintaining alignment during early weight-bearing phases and reducing the risk of secondary displacement. This increased stability is especially valuable in osteoporotic bone and comminuted fracture patterns.

Intramedullary nailing has increasingly been explored as an alternative treatment for proximal tibial fractures due to its minimally invasive nature and the potential for earlier weight-bearing and functional recovery. Compared to plate fixation, intramedullary nailing may reduce surgical exposure and lower the risk of soft tissue complications. However, achieving accurate articular reduction with intramedullary fixation can be technically challenging in complex bicondylar fracture patterns. Dual plating allows direct visualization of fracture fragments and provides stable fixation of both the medial and lateral columns, which is particularly advantageous in AO/OTA 41-C fracture patterns. Therefore, while intramedullary nailing offers certain biomechanical and soft tissue benefits, dual plating remains a reliable method for achieving stable fixation and articular congruity in complex periarticular injuries.

Early mobilization following stable fixation is essential for preventing knee stiffness and optimizing functional recovery [15]. The statistically significant improvements in knee range of motion and pain scores, confirmed by paired t-test analysis (p < 0.001), align with previous studies emphasizing the importance of rigid fixation and structured rehabilitation [16-18]. Restoring joint mobility is particularly critical in high-energy injuries, where prolonged immobilization can compromise long-term knee function and overall patient satisfaction, ultimately impacting quality of life and the ability to resume daily activities.

Radiological assessment using the RUST score confirmed progressive fracture healing. The RUST system has been validated as a reliable and reproducible tool for assessing tibial fracture union and correlates well with clinical outcomes [7,19]. The progressive increase in RUST scores during follow-up in our cohort indicates predictable healing progression following dual plating, supporting its role in achieving timely consolidation and facilitating early rehabilitation protocols with confidence in construct stability.

Complication rates in the present study were low, with superficial infections being the most common. Reported infection rates following dual plating range from 5% to 15%, depending on soft-tissue condition and timing of surgery. Adherence to the principles of biological fixation and meticulous soft-tissue handling is essential to minimize complications [20]. Careful patient selection and standardized perioperative protocols likely contributed to the favorable complication profile observed in this series, underscoring the importance of surgical expertise in managing complex fractures and implementing structured postoperative care.

Limitations

The retrospective design, lack of a comparative treatment group, relatively small sample size, and short follow-up period limit the generalizability of these findings. The moderate cohort size and absence of a formal sample size calculation may also reduce the ability to detect fewer common complications, increasing the risk of a type II statistical error. Although fracture healing was assessed using serial radiographs and the radiographic union score for tibial fractures (RUST), the six-month follow-up primarily reflects intermediate-term outcomes and may not capture late complications such as post-traumatic osteoarthritis. Furthermore, the RUST score was originally developed for diaphyseal tibial fractures treated with intramedullary fixation; its application to metaphyseal periarticular fractures treated with plate constructs represents an extrapolation beyond its original validation. Larger prospective studies with longer follow-up periods and comparative cohorts are needed to confirm these findings.

## Conclusions

Dual tibial plating appears to provide stable fixation for complex bicondylar proximal tibial fractures and is associated with high rates of fracture union and satisfactory functional outcomes, as demonstrated in this study. This technique allows for the restoration of articular alignment and supports early functional rehabilitation in appropriately selected patients. However, given the retrospective design, absence of a comparator group, and relatively short follow-up period, these findings should be interpreted with caution. Further prospective, comparative studies with longer follow-ups are necessary to confirm the long-term outcomes of dual plating in complex proximal tibial fractures.
